# Quantitative analysis of late gadolinium enhancement in hypertrophic cardiomyopathy

**DOI:** 10.1186/1532-429X-12-21

**Published:** 2010-04-07

**Authors:** Giovanni Donato Aquaro, Vincenzo Positano, Alessandro Pingitore, Elisabetta Strata, Gianluca Di Bella, Francesco Formisano, P Spirito, Massimo Lombardi

**Affiliations:** 1 MRI laboratory, Foundation G. Monasterio Regione Toscana/CNR, Pisa, Italy; 2 Clinical Physiology Institute, National Counsel of Research, Pisa, Italy; 3 Cardiovascular Department, University of Florence, Florence, Italy; 4 Clinical and Experimental Department of Medicine and Pharmacology, University of Messina, Messina, Italy; 5 Cardiology Department, Ospedali Galliera, Genova, Italy

## Abstract

**Background:**

Cardiovascular Magnetic resonance (CMR) with the late gadolinium enhancement (LGE) technique allows the detection of myocardial fibrosis in Hypertrophic cardiomyopathy (HCM). The aim of this study was to compare different methods of automatic quantification of LGE in HCM patients. Methods: Forty HCM patients (mean age 48 y, 30 males) and 20 normal subjects (mean age 38 y, 16 males) underwent CMR, and we compared 3 methods of quantification of LGE: 1) in the SD2 method a region of interest (ROI) was placed within the normal myocardium and enhanced myocardium was considered as having signal intensity >2 SD above the mean of ROI; 2) in the SD6 method enhanced myocardium was defined with a cut-off of 6 SD above mean of ROI; 3) in the RC method a ROI was placed in the background of image, a Rayleigh curve was created using the SD of that ROI and used as ideal curve of distribution of signal intensity of a perfectly nulled myocardium. The maximal signal intensity found in the Rayleigh curve was used as cut-off for enhanced myocardium. Parametric images depicting non enhanced and enhanced myocardium was created using each method. Three investigators assigned a score to each method by the comparison of the original LGE image to the respective parametric map generated.

**Results:**

Patients with HCM had lower concordance between the measured curve of distribution of signal intensity and the Rayleigh curve than controls (63.7 ± 12.3% vs 92.2 ± 2.3%, p < 0.0001).

A cut off of concordance < 82.9% had a 97.1% sensitivity and 92.3% specificity to distinguish HCM from controls. The RC method had higher score than the other methods. The average extent of enhanced myocardium measured by SD6 and Rayleigh curve method was not significant different but SD6 method showed underestimation of enhancement in 12% and overestimation in 5% of patients with HCM.

**Conclusions:**

Quantification of fibrosis in LGE images with a cut-off derived from the Rayleigh curve is more accurate than using a fixed cut-off.

## Background

Late gadolinium enhancement (LGE) cardiovascular magnetic resonance (CMR) allows detection of myocardial fibrosis in patients with hypertrophic cardiomyopathy (HCM) [1]. Previous studies showed the presence of LGE in approximately 75-80% of patients with HCM [2,3]. Quantification of LGE can be relevant for clinical purpose if we consider that myocardial fibrosis has been postulated to be an important arrhythmogenic substrate of HCM [4,5]. The association between the extent of delayed enhancement and the number of risk factor for arrhythmic event was demonstrated in younger patients[6]. However, quantification and characterization of LGE in HCM patients is difficult because of the intramyocardial patchy distribution of LGE and the presence of myocardial areas mildly enhanced, that may represent a sign of initial, non confluent, myocardial fibrosis at interstitial level (plexiform fibrosis). Plexiform fibrosis has been shown to be more evident in patients with higher arrhythmic risk [7]. Several methods assessing the extent of LGE have been proposed. In the qualitative methods the number of myocardial segments involved are visually assessed [8,9]; in semiquantitative methods an operator manually traces the contours of the enhanced area. These methods are strictly operator-dependent causing unavoidable intra- inter-observer variability. The most used quantitative method is characterised by placing a region of interest (ROI) in the normal myocardium and using a cut-off for delayed enhancement of mean intensity plus 2 standard deviations [10]. Recently mean of remote myocardium plus 6 standard deviations was used as cut-off [11]. However these methods have many limitations: the site of ROI, the assumption of a Gaussian distribution of the signal intensity in the nulled myocardium, the fixed cut-off value for identifying enhanced areas. We proposed a new method of quantification of LGE in patients with HCM without these limitations. Therefore, aim of this study was to compare methods of quantification of LGE in patients with HCM.

## Methods

### Patient population

Forty consecutive patients (30 male, mean age 40 ± 14 year) with a known diagnosis of HCM entered the study. Diagnosis of HCM was based on previous described criteria [12]. Twenty healthy normal subjects (16 males, mean age 38 ± 10 years) were enrolled as control group. As inclusion criteria controls had not familiar history of HCM or sudden cardiac death. The study was approved by the internal Ethical Committee of Clinical Physiology Institute.

### Cardiovascular Magnetic Resonance

CMR was performed using a 1.5T whole body scanner (CVi, HD release, GE Healthcare, Milwaukee, USA). An 8-element (4 anterior and 4 posterior) cardiac phased-array receiver surface coil was utilized for signal reception. Functional parameters of the left ventricle were obtained by acquiring cine short axis images from mitral valve plane to the apex with a steady-state free precession (FIESTA) pulse sequence with the following parameters: 400 mm field of view, 8 mm slice thickness, no gap, 1 NEX, 12 views per segment, TE/TR 1.6/3.2, flip angle 45°, matrix 224 × 192, and reconstruction matrix 256 × 256. End-diastolic wall thickness was measured in all the myocardial wall segment in order to confirm the diagnosis of HCM. LGE images were obtained by an Inversion Recovery Gradient Echo pulse sequence with an inversion time optimized to null signal from normal myocardium. Images were acquired 10 minutes after contrast medium (Gadolinium-DTPA, 0.2 mmol/kg) in the short axis views from the mitral valve plane to the ventricular apex. The following parameters were used: field of view 400 mm, slice thickness 8 mm, no gap between each slice, repetition time 4.6 msec, echo time 1.3, flip angle 20, field of view 360 mm, acquisition matrix 224 × 192, reconstruction matrix 256 × 256. The inversion time was optimized in order to nullify the signal from normal myocardium (range 240-300 msec).

### Myocardium signal model

It is well known that noise in magnitude MR images is Rician distributed and depends from the number of coils used in the acquisition [13,14]. Although noise distribution may be safety approximated with a Gaussian distribution if the SNR is enough high, it is not the case of the normal myocardium in LGE images where the SNR is forced to be as close as possible to zero. The probability density function (PDF) of Rician distributed noise is quite complex. However, if the myocardium is perfectly nulled the signal PDF follows a more simple generalized Rayleigh distribution:

where m represents the measured signal, K the number of phased array coils, and the unit step function ε (m) is used to indicate that the expression is valid only for nonnegative m values. σ is the standard deviation of noise that can be estimated from image background.

**Figure 1:**
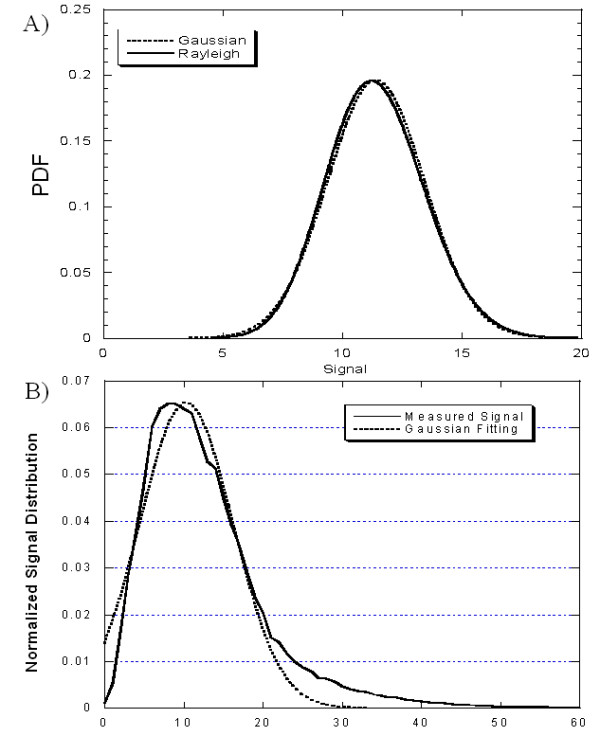
**Statistical distribution of signal in normal myocardium**. A) shape of the generalized Rayleigh PDF and of the Gaussian PDF for a background SD of 2.05 as measured in the present study in normal patients and for K = 8 phased array coils; B) measured distribution of the myocardial signal in normal patients as a normalized histogram and gaussian distribution.

If phased array coils are used, Rician noise distribution depends from the number of elements and become more similar to a standard Gaussian distribution when the number of elements increases. An 8-elements coil as the one used in the present study produce a noise distribution that is almost Gaussian [15]. Figure 1A shows the shape of the generalized Rayleigh PDF and of the Gaussian PDF for a background SD of 2.05 as measured in the present study in normal patients and for K = 8 phased array coils. The two distributions are very similar, so that for the acquisition procedure described in this study the noise distribution can be safety considered as Gaussian-like in normal patients. But, if the myocardium is not perfectly nulled, due the multiplicative nature of the Rician distributed noise [16] both mean and SD of the measured signal become different from the background. Similarly the distribution of the shape in myocardium can also be modified by overload of contrast medium as in presence of fibrosis.

A normalized histogram of the signal intensities in ROIs was evaluated for each normal subject and HCM patient. A curve of the distribution of pixel intensity in the whole myocardium was obtained. Each curve distribution was normalized respect to the total number of pixel measured and expressed in percentage. Figure 1B shows the measured distribution of the myocardial signal in normal patients as a normalized histogram, obtained by the methodology described in the next paragraph. The best Gaussian fitting obtained by the Levenberg-Marquardt fitting algorithm is showed as well. The measured fitting error is 3.5%. The measured and Gaussian estimate distribution are significantly different (t = 3.943, p < 0.0001).

### Image analysis

**Figure 2:**
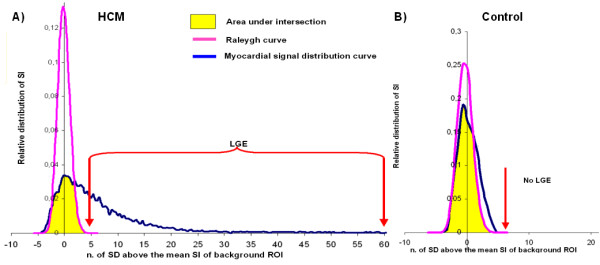
**Overlapping between the Rayleigh curve and the measured curve of distribution of signal intensity in a patient with HCM and in a control**. The yellow area is the intersection between the curves. The concordance between the curves was measured as the ratio between the area of intersection and the area under the Rayleigh. In HCM patients (A) the two curves were very different and LGE was defined as the myocardial signal higher than the maximal signal of the Rayleigh. In the control (B) no LGE was detected.

DICOM image were converted in a 8-bit scaling using a fixed minimum-to-maximum windowing algorithm in order to obtain a fixed grayscale of pixel intensity from 0 to 255 signal intensity. Endocardial and epicardial contours in each image were manually traced to identify left ventricular tissue in each image. Within the whole myocardial mass the signal intensity was measured and a curve of distribution of myocardial signal intensity was obtained. A square 25 × 25 mm region of interest (ROI) was placed in the background of the image, near the patient's thoracic wall (background-ROI). Mean SI and SD were measured in the background- ROI. An other ROI was placed in a portion of myocardium of LV evaluated as normal (myocardial-ROI). Using the SD of signal intensity measured in the background-ROI, a Rayleigh curve was obtained. This Rayleigh curve was considered the ideal curve of distribution of signal intensity of a perfectly nulled myocardium because signal in the background-ROI was due to only the signal noise and the ratio between the signal intensity measured in nulled myocardium and the signal intensity of the background tends to 1 [14]. The curve of distribution of myocardial signal intensity was overlapped to the Rayleigh curve. The concordance between the two curve was measured as the area of the intersection between them (figure 2A-B) and it was expressed as a percentage of the area under the Rayleigh curve.

We compared two approaches to quantify the extent of higher and mild enhancement in patients with HCM. In the first approach (SD2 method), mean SI and SD measured in the myocardial-ROI were used: higher enhancement was defined as myocardium with SI >mean of myocardial-ROI plus 2 SD.

In the second approach (SD6 method), mean SI and SD measured in the myocardial-ROI were used: higher enhancement was defined as myocardium with SI >mean of myocardial-ROI plus 6 SD.

We proposed a method of quantification of LGE using the Rayleigh curve (RC method). Briefly, as evidenced in figure 2A, LGE was defined as myocardium having signal intensity > the maximal signal intensity measurable in the Rayleigh curve (red line).

Using the different thresholds of signal intensity derived from the three methods simplified parametric maps were created from each original LGE image depicting with different colours areas of normal myocardium and enhanced myocardium.

In order to compare the two methods of analysis the parametric map obtained from each methods was compared with the respective original image by three independent expert investigators (GDA, ES, GD) and a score ranging from 1 to 4 (1 = not suitable, 2 = sufficient, 3 = good, 4 = excellent) was given. The mean score for each method was calculated in each patient. If a score of ≥ 3 was not assigned, the investigators specified if an underestimation of overestimation was performed by the methods by the visual comparison of the original image and the respective parametric map. Overestimation was defined when the area of enhanced myocardium (pink region) in the parametric map exceeded the visual appreciation of the enhanced myocardium in the original image. At the opposite, underestimation was defined when the area of enhanced myocardium depicted in the parametric map was smaller than the corresponding area of enhancement in the original image.

The extent of higher enhancement and mild enhancement in percentage respect to the ventricular mass was automatically measured using both the methods of analysis in each patient.

Contrast-to-noise ratio and signal-to-noise ratio were measured in the non-enhanced myocardium in order to evaluate the myocardium nulling. Contrast-to-noise ratio was measured with the formula: 2 × (mean *myoI *- mean *bkI*)/(SD *myoI *+ SD *bkI*), where *myoI *is the mean signal intensity of an ROI placed in non-enhanced myocardium and *bkI *is the signal intensity of an ROI placed in the background [17]. By the above formula, in perfectly nulled myocardium Contrast-to-noise ratio should be equal to 1.

### Statistical analysis

All data were analysed with the use of JMP version (4.0). The inter-observer variability for the assigned score was evaluated by the Kappa test. Data are presented as continue variable and proportions (percentages). Continuous variables are expressed as mean ± 1 SD. Two-way analysis of variance (ANOVA) analysis was used to compare tysis. A p value < 0.05 was considered statistically significant. A receiver operating characteristics (ROC) curve was used to evaluate the sensitivity and specificity of the concordance between the measured distribution curve of signal intensity and the Rayleigh curve to differentiate between patients with HCM and the other groups (control group and athletes).

## Results

The clinical variables of the patients with HCM and controls are showed in table 1. The LV mass index and the maximal end-diastolic wall thickness were significantly higher in patients with HCM than in controls.

**Table 1:** Summary of clinical data for the entire population

Clinical Variables	HCM	p value	Controls
Number	40		20
Male (n)	30(75%)	0.9	16(80%)
Age (years)	40 ± 14	0.6	38 ± 10
CMR findings:			
LV mass index (g/m^2^)	107 ± 36	<0.0001	64 ± 14
EDVi (ml/m^2^)	77 ± 17	0.8	78 ± 12
Maximal ED thickness (mm)	19 ± 5	<0.0001	9 ± 4
Ejection fraction (%)	68 ± 10	0.24	65 ± 8

SD, standard deviation; CMR, cardiovascular magnetic resonance; LV, left ventricle; EDVi, End Diastolic Volume index; ED, End Diastolic.

**Figure 3:**
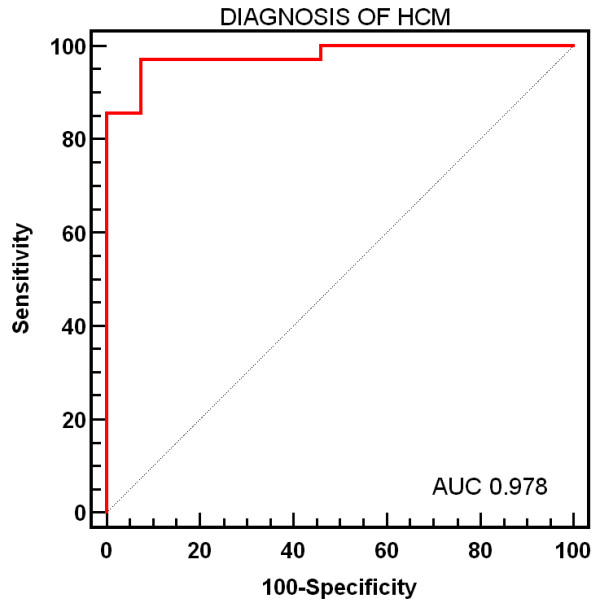
**ROC curve of Rayleigh curve for the diagnosis of HCM**. ROC curve evaluating the sensitivity and specificity of the concordance between the measured curve of distribution of signal intensity and the Rayleigh curve for the diagnosis of HCM.

A total of 483 images were analysed in HCM patients and 212 images in normal patients. Mean contrast-to-noise ratio evaluated between normal myocardium and background was not different in HCM patients and normal patients (1.09 ± 0.9 vs 1.29 ± 01.2, p = ns). By a visual qualitative analysis observers were able to identify higher enhancement in 32 (80%) patients with HCM. At visual analysis mild enhancement was not detected in any patients in the normal group. Patients with HCM had lower concordance between the measured curve of distribution of signal intensity and the Rayleigh curve than normal patients (63.7 ± 12.3 vs 92.2 ± 2.3%, p < 0.0001). At ROC analysis (figure 3) the concordance between the measured distribution of SI curve and the Rayleigh curve showed a sensitivity of 97.1% and a specificity of 92.3% to distinguish between patients HCM from controls (cut-off 82.9%, area under the curve of 0.978).

**Figure 4:**
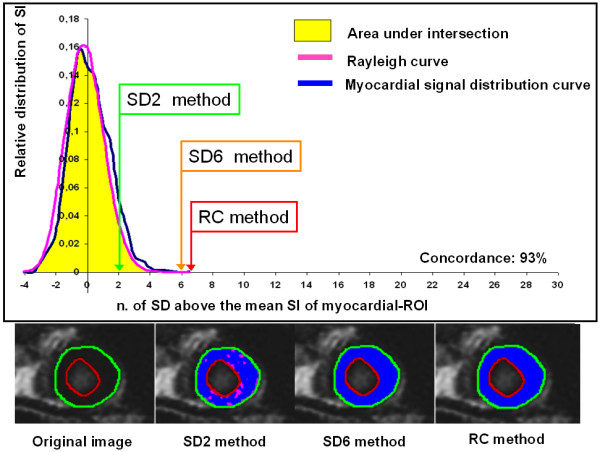
**Comparison between the methods in a control**. Analysis of an original LGE image of a control patient. The upper panel shows the relative distribution of pixel intensity of myocardium (blue line) and the Rayleigh curve derived from the signal of the background-ROI (pink line). In this patient there is a good overlap between the two curves (concordance 93%). The thresholds of SD2, SD6 and RC methods are evidenced. In the inferior panels in left to right order are showed the original LGE image contoured, the parametric map derived from the SD2 method, from the SD6 method and from the RC method. In this case SD2 committed a large overestimation of enhancement as evidenced both in the graph and in the parametric map.

**Figure 5:**
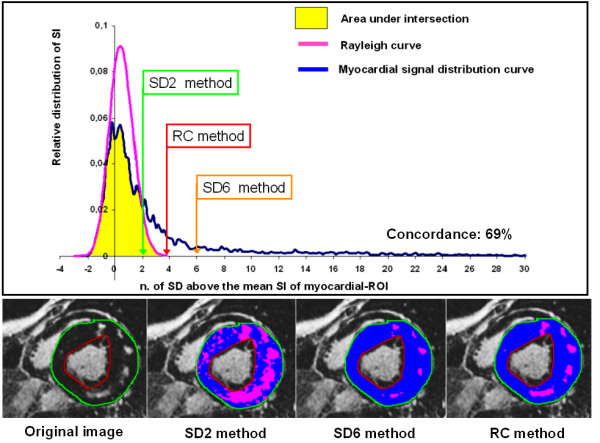
**Comparison between the methods in a patient with HCM**. Analysis of an original LGE image of a patient with HCM. The upper panel shows the relative distribution of pixel intensity of myocardium (blue line) and the Rayleigh curve derived from the signal of the background-ROI (pink line). The good overlap between the two curves was low (concordance 69%) demonstrating a significant difference between the ideal curve of distribution (Rayleigh) and the measured curve. The thresholds of SD2, SD6 and RC methods are evidenced. In the inferior panels in left to right order are showed the original LGE image contoured, the parametric map derived from the SD2 method, from the SD6 method and from the RC method. In this case SD2 committed a large overestimation of enhancement and SD6 a slight underestimation as evidenced in the graph and judged by the investigators by the comparison between the parametric map and the original image.

Inter-observer variability for the score assigned was good (observer 1-2 κ = 0. 79, observer 1-3 κ = 0.82, observer 2-3 κ = 0.77). The SD2 method showed the lowest mean score compared to the other methods in the HCM group and in controls (table 2). SD2 method committed an "overestimation" in 38 patients with HCM (95%) and in and in all the controls (figure 4 and 5). The SD6 method had higher score than the SD2 method in HCM group and in controls. SD6 method had significant lower score than RC method in HCM group. SD6 and RC method had not significant different mean score in controls.

**Table 2:** Method comparison in HCM and controls

Variable	RC	p value	SD2	p value	SD6	p value (vs RC)
**HCM:**						
Mean score	3.75 ± 0.4	<0.0001	1.45 ± 0.5	<0.0001	3.12 ± 0.5	<0.01
Extent of enhancement (% mass)	3.66 ± 5.1	<0.0001	12.2 ± 5.4	<0.0001	5.52 ± 7.2	0.12
**Controls:**						
Mean score	3.8 ± 0.5	<0.0001	1.05 ± 0.5	<0.0001	3.6 ± 0.5	0.28
Extent of enhancement(% mass)	0.005	<0.0001	5.4 ± 5.9	<0.0001	0.02	0.7

% mass, extent of enhancement was expressed as percentage of left ventricular mass

SD6 methods committed overestimation in 2 (5%) patients with HCM and in 1(5%) patients in the control group, underestimation in 5 (12.5%) patients with HCM.

RC method did not commit "underestimation" in patients with HCM but showed "overestimation" in 1 patients (5%) in the control group.

As evidenced in table 2, the average extent of enhanced myocardium was significantly higher in SD2 method than the other methods while no significant differences were found between SD6 and RC methods.

## Discussion

In the present study, three different quantitative methods to detect and quantify enhanced myocardium in LGE images in HCM patients have been compared. The results showed significant differences between the methods. This may result from two main factors: 1) the threshold values of SD above the mean signal intensity of the reference ROI; 2) the site of the reference ROI.

The approach of 2 SD and reference ROI within the myocardium (SD2 method) was largely used as semi-quantitative method for quantification of LGE in literature. In our study, this method showed a larger overestimation of the extent of LGE in comparison with the method we proposed, and resulted unsuitable for an operator independent quantification of LGE. As shown in figures 4 and 5, using as threshold 2 SD above the mean intensity a large amount of normal myocardium is considered as pathologic committing a large overestimation, linked to both the reduced number of SD used and to the low value of the SD found in the remote myocardium. In fact, this method does not consider that the distribution of signal intensity is much closer to a Rician distribution rather then to a Gaussian fitting as showed in methods. Similar result were found by Bodarenko et al.[18]. In that study the extent of delayed enhancement in myocardial infarction was measured using different cut-off (from >2 SD to >6 SD above the mean signal intensity) derived from a ROI placed in remote myocardium. Also in myocardial infarction large overestimation of infarct size was found using a cut-off of >2 SD above the mean. Finally, in HCM patients the detection of area of "normal" myocardium can be unsuccessful due to the presence of large amount of plexiform fibrosis. Instead, choosing ROI in the background had several advantages: the ROI size can be bigger increasing the statistic counting; signal artefacts originated by the partial volume effect and unnoticed presence of fibrosis in myocardium are avoided; finally signal intensity distribution in the image background and in perfectly nulled myocardium are exactly the same.

A cut off of >6 SD above the mean of remote ROI was used in previous studies [11,19] as fixed threshold to quantify enhanced myocardium. In this study this SD6 method had higher mean score than the SD2 method in HCM and control groups. Yet, mean score of SD6 method was not significant different to RC method in control group but it was significant lower in HCM group.

Although the mean extent of enhanced myocardium measured by SD6 method was not significantly different from the extent measured by the RC methods, SD6 committed overestimation of enhanced myocardium in 12% and underestimation in 5% of patients with HCM.

We proposed a more complex method of quantification combining the use of Rayleigh curve and the measured curve of distribution of signal intensity. Rayleigh curve, created using the SD of the background ROI, was considered a model of the ideal distribution of signal intensity in a perfectly nulled myocardium. In fact, when myocardium is perfectly nulled, its signal derives only from the signal noise and it should be absolutely identical to the background signal. Thus, in perfectly nulled myocardium the signal intensity ratio between background and myocardium evaluated by the contrast-to-noise ratio should be equal to 1. It was previously demonstrated that the distribution of noise in of MRI image is Rician and it could be approximated to a Rayleigh curve [13-17]. Because the signal in the nulled myocardium should be only the noise, its curve of distribution of signal intensity should necessarily resemble a Rayleigh curve. Thus, the higher the concordance between the Rayleigh curve calculated from the signal of background and the curve of distribution of myocardial signal intensity, the higher is the probability that the myocardium is normal. Thus an enhanced, probably diseased, myocardium have a curve of signal distribution very different from the Rayleigh curve. In this study patients with HCM had a mean concordance of 64%, whereas controls had a mean concordance of 92% between Rayleigh and distribution of SI curve.

Furthermore, regions of myocardium with signal intensity higher than the maximal value of the Rayleigh curve have very high probability to be enhanced. With this assumption, any signal intensity higher than the maximal signal intensity derived from the Rayleigh curve was considered pathologic (enhanced myocardium). This new method for quantification of enhanced tissue in LGE images had higher average score than the other methods without committing any over- or underestimation in the HCM group.

Results of this study suggest that the measurement of the degree of concordance between the Rayleigh curve and the measured curve of distribution of signal intensity could be a valuable approach to distinguish between HCM and controls.

The higher the involvement of myocardium, the lower the concordance between the ideal and measured curve. A cut off of 82.9% of concordance had higher sensitivity and specificity to distinguish between HCM and controls. This could be potentially useful for the differential diagnosis between HCM and other causes of left ventricular hypertrophy as athletes heart, acromegaly. The value of this parameters in the clinical setting should be evaluated by further studies.

Another advantage of this method respect to the others is the absence of a fixed cut-off. In fact the cut-off derived from the Rayleigh curve is variable and depends from the mean signal intensity and SD of the background-ROI. Thus, when the signal intensity and SD of the background is higher because of a higher signal noise, the generated Rayleigh curve modifies its morphology and the cut-off is shifted to higher value. In this way the risk of under- and overestimation of the enhanced myocardium is minimized. Contrarily, using a fixed cut-off, when the images are noised the detection and the quantification of the enhancement myocardium could be underestimated in HCM because the remote myocardium could have higher signal intensity due to the higher noise.

In our study DICOM images were normalized to a 8 bit scale. This conversion allowed to obtain a curve of distribution of pixel intensity in a fixed scale from 0 to 255 in every patients. A larger grayscale (i.e. 16 bits) of pixel probably leads to more variable mean intensity and to a larger SD in ROI. However, since the curve of distribution of intensity is larger in DICOM but with a preserved proportionality the final result in image analysis is similar. Moreover, whereas the grayscale of signal intensity in DICOM image changes from patient to patient due to difference in shimming and magnetic field distortion, the conversion to a 8 bit scale permits to avoid this disadvantage and to compare signal histograms in all the patients independently from the adopted method. Using a "fixed" minimum-maximum algorithm and transforming the grayscale of intensity from 0 to 255, differences of windowing are nulled. In fact the signal intensity information stored in DICOM image were transformed in all the patients in a common grayscale: to the maximal signal intensity was always assigned a value of 255, to the minimal signal intensity a value of 0. Thus, windowing ca not influence the analysis of images and cannot modify the value of signal intensity of the voxel.

In this study LGE images were acquired with a fixed matrix and slice thickness. Different result in terms of quantification of myocardial enhancement could be found using different protocol with different slice thickness, matrix and pulse sequence. LGE images in this study showed a very low myocardial contrast-to-noise ratio as result of an appropriate choice of inversion time and suppression of myocardial signal. A worse myocardial suppression could permit to obtain higher mean signal intensity probably alter these results.

## Conclusions

In normal patients the distribution of signal intensity in a perfectly nulled myocardium is very similar to a Rayleigh curve. In HCM the concordance between the measured curve of distribution and the Rayleigh is significantly lower. Using the maximal value of signal intensity found in the Rayleigh curve as threshold is more effective than methods that imply a fixed cut-off (SD2 and SD6 methods) for the quantification of enhanced myocardium in LGE images.

## Competing interests

The authors declare that they have no competing interests.

## Authors' contributions

GDA conceived and designed the study, gave a major contribution to the draft of the manuscript and its revision, and was involved in data analysis and statistical calculation. VP gave a contribution in conception of methods and in drafting the manuscript. AP was involved in revision of manuscript.

ES gave a contribution for image and data acquisition and was involved in analysis of data. GD gave a contribution of data acquisition and data analysis. FF carried out subject's recruitment and results interpretation. PS carried out subject's recruitment and revision of manuscript. ML gave the final approval of the manuscript.
